# Catalytic oxidation of arsenite and reaction pathways on the surface of CuO nanoparticles at a wide range of pHs

**DOI:** 10.1186/s12932-018-0058-3

**Published:** 2018-06-22

**Authors:** Lingqun Zeng, Biao Wan, Rixiang Huang, Yupeng Yan, Xiaoming Wang, Wenfeng Tan, Fan Liu, Xionghan Feng

**Affiliations:** 1 Key Laboratory of Arable Land Conservation (Middle and Lower Reaches of Yangtze River), Ministry of Agriculture College of Resources and Environment, Huazhong Agricultural University, Wuhan, 430070 China; 2 School of Earth and Atmospheric Sciences, Georgia Institute of Technology, 311 Ferst Dr, Atlanta, GA 30324-0340 USA

**Keywords:** CuO NPs, As(III), Oxidation, Adsorption, Surface catalysis

## Abstract

Recently, the wide application of CuO nanoparticles (NPs) in engineering field inevitably leads to its release into various geologic settings, which has aroused great concern about the geochemical behaviors of CuO NPs due to its high surface reactivity and impact on the fate of co-existing contaminants. However, the redox transformation of pollutants mediated by CuO NPs and the underlying mechanism still remain poorly understood. Here, we studied the interaction of CuO NPs with As(III), and explored the reaction pathways using batch experiments and multiple spectroscopic techniques. The results of in situ quick scanning X-ray absorption spectroscopy (Q-XAS) analysis verified that CuO NPs is capable of catalytically oxidize As(III) under dark conditions efficiently at a wide range of pHs. As(III) was firstly adsorbed on CuO NPs surface and then gradually oxidized to As(V) with dissolved O_2_ as the terminal electron acceptor. As(III) adsorption increased to the maximum at a pH close to PZC of CuO NPs (~ pH 9.2), and then sharply decreased with increasing pH, while the oxidation capacity monotonically increased with pH. X-ray photoelectron spectroscopy and electron paramagnetic resonance characterization of samples from batch experiments indicated that two pathways may be involved in As(III) catalytic oxidation: (1) direct electron transfer from As(III) to Cu(II), followed by concomitant re-oxidation of the produced Cu(I) by dissolved O_2_ back to Cu(II) on CuO NPs surface, and (2) As(III) oxidation by reactive oxygen species (ROS) produced from the above Cu(I) oxygenation process. These observations facilitate a better understanding of the surface catalytic property of CuO NPs and its interaction with As(III) and other elements with variable valence in geochemical environments.

**Electronic supplementary material:**

The online version of this article (10.1186/s12932-018-0058-3) contains supplementary material, which is available to authorized users.

## Introduction

Compared with its micro or bulk counterparts, CuO nanoparticles (NPs) possesses better optical, semiconductive and surface reactive properties, and is thus widely used for the production of ceramics, glass and pigments, catalysts, battery anodes, and antimicrobial agents [1–5]. Due to the increasing application of CuO NPs in industrial fields, its release and geochemical behaviors in the environment have aroused great concern [6–9]. Its potential toxicity to the organisms has been studied in detail [10–14]. Apart from the direct biotic effects, CuO NPs might also impact the mobility, transformation and toxicity of the co-existing contaminants through (de)sorption, redox and catalytic reactions [15–17]. However, little is known about the interaction of CuO NPs with redox-sensitive contaminants and the underlying reaction mechanism.

Arsenic (As) is the most common heavy metal in natural waters primarily in the forms of inorganic arsenate [As(V)] and arsenite [As(III)] [18]. Redox processes on the surface of oxide minerals largely determine the speciation of As [19–21]. Compared with As(V), As(III) has higher toxicity, solubility and mobility [22].

CuO NPs is an excellent adsorbent to remove As(III) from water due to its large specific surface area and high point of zero charge (PZC) [17, 23–25]. In addition, X-ray photoelectron spectroscopy (XPS) analysis suggested that As(III) could be oxidized on CuO NPs surface, which remarkably enhances the immobility of As in the form of As(V) [17, 24]. A previous study has proposed the direct electron transfer from CuO NPs to As(III), which leads to As(III) oxidation [24]. Given that XPS measurement is performed under a high vacuum condition, more solid evidences are needed to verify As(III) oxidation on the surface of CuO NPs.

Furthermore, it has been reported that reactive oxygen species (ROS) is involved in the oxidation pathway of organic matter [26–28]. For example, remarkable amounts of Cu^+^/Cu^2+^ and H_2_O_2_ were formed in zero-valent copper (ZVC) acidic system due to the corrosive dissolution of ZVC and the concurrent reduction of oxygen, which lead to highly efficient oxidation of diethyl phthalate under aerobic atmosphere condition [26]. In addition, a synergistic effect of Fe(II) and copper oxide (CuO) was observed on the degradation of acetaminophen in the presence of O_2_, and the resulting Cu(I) significantly accelerated the destruction of acetaminophen by serving as an electron-mediator between the adsorbed Fe(II) and O_2_ to produce ROS [15]. Therefore, it is possible that ROS might be produced through the activation of O_2_ on the surface of CuO NPs, which leads to the catalytic oxidation of As(III).

Thus, the objectives of this study are to (i) in situ confirm whether As(III) oxidation occurs on the surface of CuO NPs under dark condition; (ii) determine the effects of geochemical parameters [such as pH, As(III) concentration and O_2_] on As(III) adsorption and oxidation by CuO NPs; (iii) elucidate the adsorption and oxidation pathways of As(III) on the surface of CuO NPs at a wide range of pH values under dark condition. To achieve these objectives, batch experiments and spectroscopic analysis were performed. Time-resolved quick scanning X-ray absorption spectroscopy (Q-XAS) and electron paramagnetic resonance (EPR) spectroscopy were used to in situ measure the species of As and ROS, respectively. XPS spectroscopy was also used to determine the changes in oxidation state of Cu and As on the surface of CuO NPs after the reaction.

## Experimental

### Preparation and characterization of CuO NPs

CuO NPs was prepared via a previously reported method [5, 29]. The synthesized CuO NPs contained no impurity phases as examined by powder X-ray diffraction (PXRD), Fourier-transformed infrared (FTIR) spectroscopy and transmission electron microscopy (TEM) analyses. The specific surface area was determined by Brunauer–Emmett–Teller (BET) N_2_ adsorption method. Additional details of analytical procedures and characterization results are provided in Additional file 1: Figures S1, S2, S5.

### As(III) adsorption and oxidation kinetics

The adsorption-oxidation experiments were conducted by the reaction of 0.2 g CuO NPs with 200 mL 10 mg L^−1^ As(III) at pH 6, 9, and 11, respectively. The background electrolyte was 0.01 M NaCl solution. The CuO NPs suspensions were agitated by magnetic stirring at 10 Hz. Solution pH was measured using a pH meter (FE20, Mettler-Toledo) and manually adjusted to desired pH values ± 0.1 using 0.1 M HCl and 0.1 M NaOH. To examine the effect of dissolved oxygen (O_2_) on the oxidation of As(III), the reaction solution was purged by N_2_ before and during the reaction. At the selected reaction time, 5 mL suspension was filtered through 0.22 μm Millipore membrane to analyze the concentrations of As(III) and As(V) in the supernatant. The wet solids on the membrane were dissolved by 1 mL 1 M HCl to analyze the amount of As(III) and As(V) adsorbed onto CuO NPs surface. Another 5 mL suspension was directly dissolved by 1 M HCl (1 mL) to analyze the total amount of As(III) and As(V) in the suspension. All experiments were performed in triplicates. The As(V) concentration was measured by the molybdene blue method [30]. The total As was determined by hydrate generation atomic fluorescence spectrometry (HG-AFS) (AFS-230E) [31]. Additionally, the volume of 0.1 M NaOH consumed in open system and N_2_ system at pH 11 was recorded by automatic titrator (Metrohm 907 Titrando). All As(III) adsorption and oxidation experiments were carried out in reactors covered with aluminum foil to avoid the effect of light.

### Effect of pH and initial As(III) concentration on As(III) adsorption and oxidation

To investigate the effect of pH values on the species distributions of As(III) and As(V) in solution and on CuO NPs surface, As(III) adsorption and oxidation were evaluated at pH 5, 6, 7, 8, 9, 10 and 11 by adjusting to the desired pH values ± 0.1 using 0.1 M HCl and 0.1 M NaOH. The experiments were carried out in 50-mL polyethylene centrifuge tubes by mixing 0.025 g of CuO NPs with 25 mL of 0.01 M NaCl containing fresh 10 mg L^−1^ As(III). To investigate the effect of initial As(III) concentration on As(III) adsorption and oxidation, the experiments were performed at initial As(III) concentrations ranging from 0.5 to 80 mg L^−1^ at pH 8 and 11. The reaction suspensions were equilibrated by shaking at 200 rpm and at 25 °C for 48 h. During the reaction, the pH of each batch sample was adjusted to the designed pH ± 0.05 at 1, 6, 12, 24, 36 and 48 h, respectively. After 48 h of reaction, the species distributions of As(III) and As(V) in solution and on CuO NPs surface were analyzed with the same procedures as described in **“**As(III) adsorption and oxidation kinetics**”**.

### Quick Scanning K-edge X-ray absorption spectroscopy (Q-XAS) of As

Q-XAS was used to in situ investigate the changes in oxidation state of As with increasing reaction time. The Q-XAS spectra were measured at room temperature on the 1W2Bbeamline at the Beijing Synchrotron Radiation Facility(BSRF). Considering the detect limitations (≥ 100 mg L^−1^ for As) of Lytle prober, higher concentrations of A_S_(III) (150 mg L^−1^) and CuO NPs (5 g L^−1^) were used for the in situ Q-XAS measurement. The reaction was performed in 50-mL polypropylene reaction vessels (see Additional file 1: Scheme S1), into which a 1 × 3 cm slit was cut and sealed with Kapton tape, backed with Kapton film to prevent the interaction between the tape and suspension. The As K-edge XAS spectra was collected immediately when As(III) solution was added into the suspension. The measurement time for each XAS spectrum is 1 min and the total time for the in situ XAS experiment is 8 h. Additional experimental details are described in Additional file 1: S3.

### X-ray photoelectron spectroscopic (XPS) analysis

To determine the oxidation state of As and Cu on the surface of CuO NPs, the samples prepared from the reaction of 0.05 g CuO with 50 mL of 10 mg L^−1^ As(III) for 12 h were measured with XPS using a monochromatic Al Kα X-ray source (VG Multilab 2000 X-ray photoelectron spectrometer). The scans were carried out in an energy range of 1100–5 eV to obtain XPS spectra for C1 s, Cu 2p, and As 3d. The position of binding energy was corrected by fixing the C1 s peak at 284.6 eV using the Advantage v6.5 software.

### Electron paramagnetic resonance (EPR) spectroscopy

For the EPR experiment, 50 mL reaction suspension was prepared to contain 1 g L^−1^ CuO NPs and 10 mg L^−1^ As(III) at pH 11 over 2 h under stirring. At the selected time, 3 aliquots of 2 mL suspension were sampled for the detection of ROS speciation. The detection methods and procedures for different ROS species are described in Additional file 1: S4.

## Results and discussion

### As(III) adsorption and oxidation kinetics

**Fig. 1:**
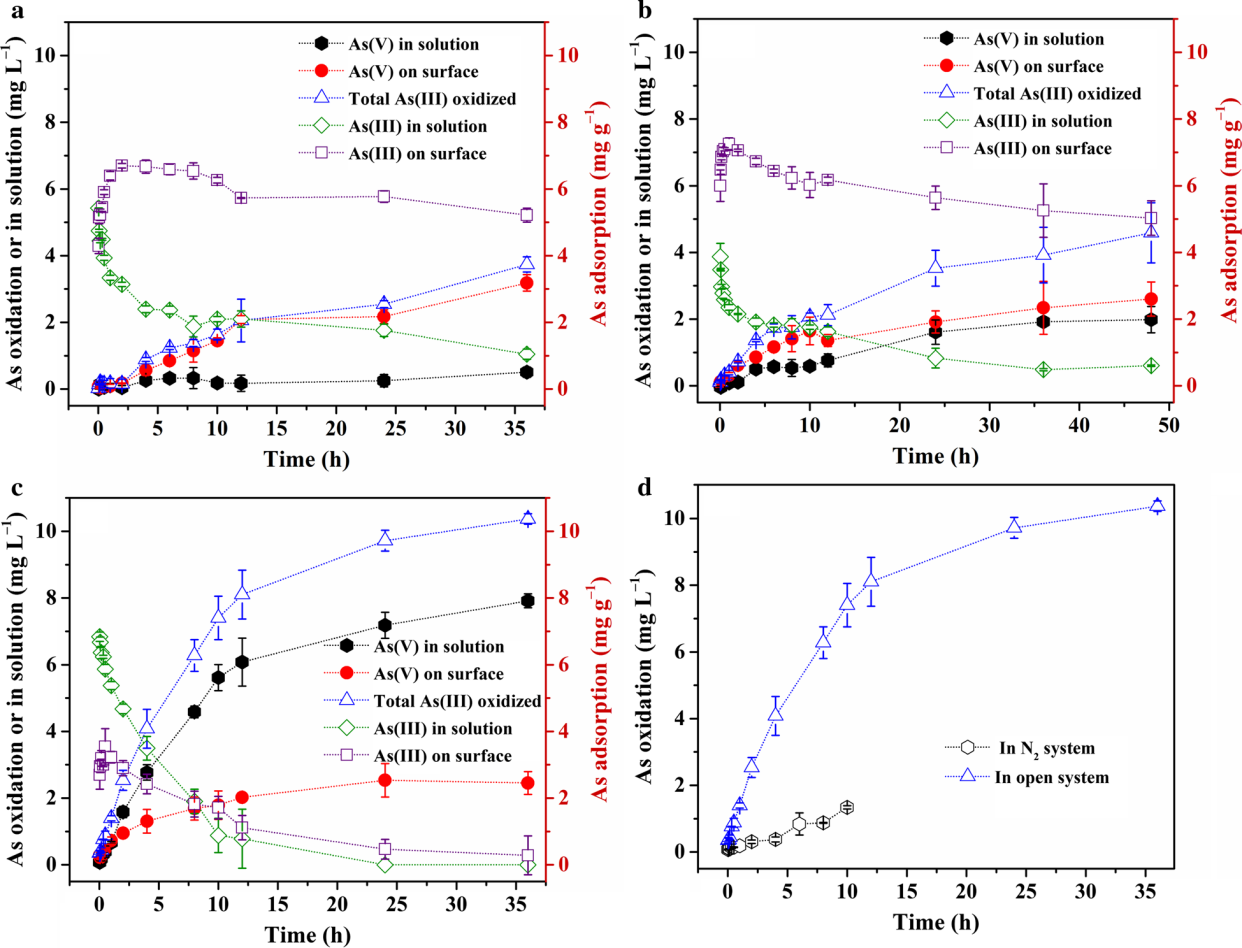
Kinetics of As(III) oxidation on CuO NPs surface at pH 6 (**a**), pH 9 (**b**), and pH 11 (**c**) in the open system, and pH 11 in the N_2_ atmosphere (**d**). These figures showed the concentration distribution of As(III) and As(V) species in solution, on CuO NPs surface, and total oxidized As(III) amount during the As(III) oxidation reaction

Adsorption and oxidation of As(III) occurred while the rate of adsorption was relatively higher at the initial reaction stage (Fig. 1). The sum of surface-adsorbed As(III) and As(V) could indicate the adsorption capacity of CuO NPs, which was about 8.39 mg g^−1^ and 7.95 mg g^−1^ respectively at pH 6 and 9 and much lower at pH 11 (2.73 mg g^−1^). Surface adsorption of As(III) reached the maximum within 2 h at all three pHs, and then gradually decreased. In contrast, both surface-adsorbed and solution As(V) gradually increased at all pHs, except for solution As(V) at pH 6, which remained consistently low over time. Although the surface adsorption of As(V) was consistent among all three pHs, the amount of solution As(V) increased with increasing pH, suggesting a higher oxidation efficiency at high pH (please also refer to **“**Effect of pH and initial As(III) concentration on As(III) adsorption and oxidation**”**. In addition, both adsorbed and solution As(III) decreased as pH increased, revealing a high oxidation efficiency and low adsorption affinity of As(III) at high pH.

To determine the rate of As(III) oxidation by CuO NPs, the concentration of As(III) remaining in the system was fitted using first-order kinetic equation (Additional file 1: Fig. S2). The fitted rate constants were 0.013, 0.014, and 0.14 h^−1^ at pH 6, 9, and 11, respectively (Table 1). The oxidation rates at pH 6 and 9 were very close to each other, but were about an order of magnitude smaller than that at pH 11. The oxidation kinetics under N_2_ atmosphere at pH 11 were also measured to investigate the influence of O_2_ level. The oxidation rate (0.012 h^−1^) and oxidation extent were both lower under N_2_ than under O_2_ (Fig. 1d and Additional file 1: Fig. S2d), implying that O_2_ could be the terminal electron acceptor for As(III) oxidation (**for details refer to** **“**XPS analyses**”**). The lower rate and extent of As(III) oxidation under N_2_ condition could be ascribed to the direct electron transfer from a small amount of As(III) to surface Cu(II) sites on CuO NPs.

**Table 1:** Apparent, first-order rate constants determined from batch and Q-XAS experiments

	Experiment type	Time period (h)	No. of data points	k (h^−1^)	R^2^
In air	As(III)—Batch-pH 6	36	14	0.013	0.96
	As(III)—Batch-pH 9	48	14	0.014	0.93
	As(III)—Batch-pH 11	36	12	0.14	0.99
In N_2_	As(III)—Batch-pH 11	10	11	0.012	0.96
In air	As(III)—Q-XAS-pH11	8	376	0.030	0.66

The rate constants of As(III) depletion were determined by linear regression analysis of the noted time-periods for the plots in Additional file 1: Fig. S2

### Effects of pH and initial As(III) concentration on As(III) adsorption and oxidation

**Fig. 2:**
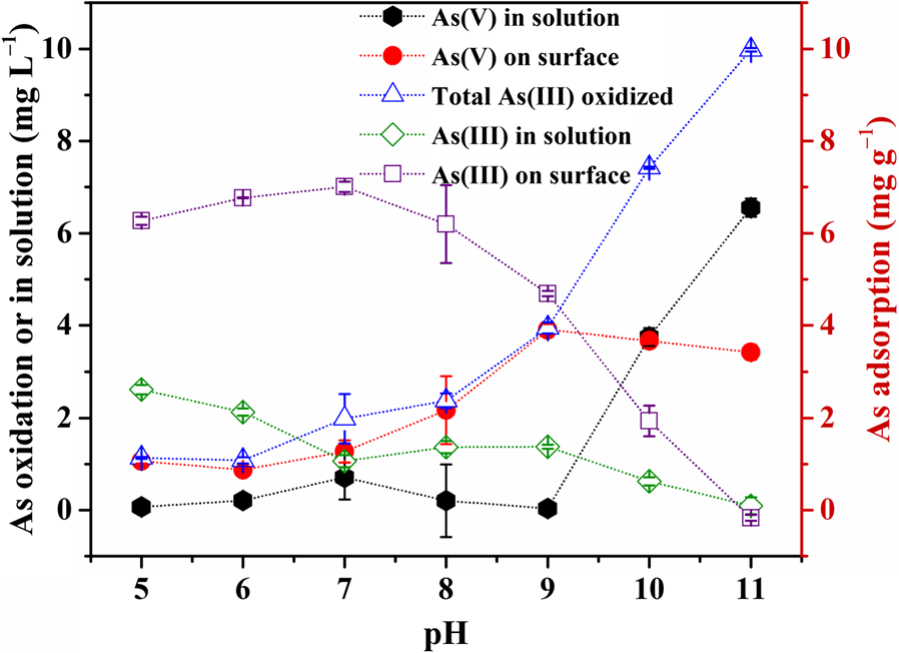
The concentration distribution of As(V) and As(III) in solution, on CuO NPs surface and total oxidized As(III) amount during the As(III) oxidation reaction at various pHs

The phase distributions of As(III) and As(V) at different pHs over 48 h are shown in Fig. 2. The total amount of As(III) oxidized by CuO NPs increased with increasing pH, particularly under alkaline condition. More specifically, the total amount of oxidized As(III) increased from 1.13 to 2.37 mg L^−1^ as pH increased from 5 to 8, and sharply rose from 3.94 to 9.98 mg L^−1^ with pH increasing from 9 to 11. In addition, most As(V) was partitioned on CuO NPs surface at pH below the PZC (9.2), but migrated into the aqueous phase at pH above the PZC, due to electrostatic repulsion between As(V) and negatively charged surface.

**Fig. 3:**
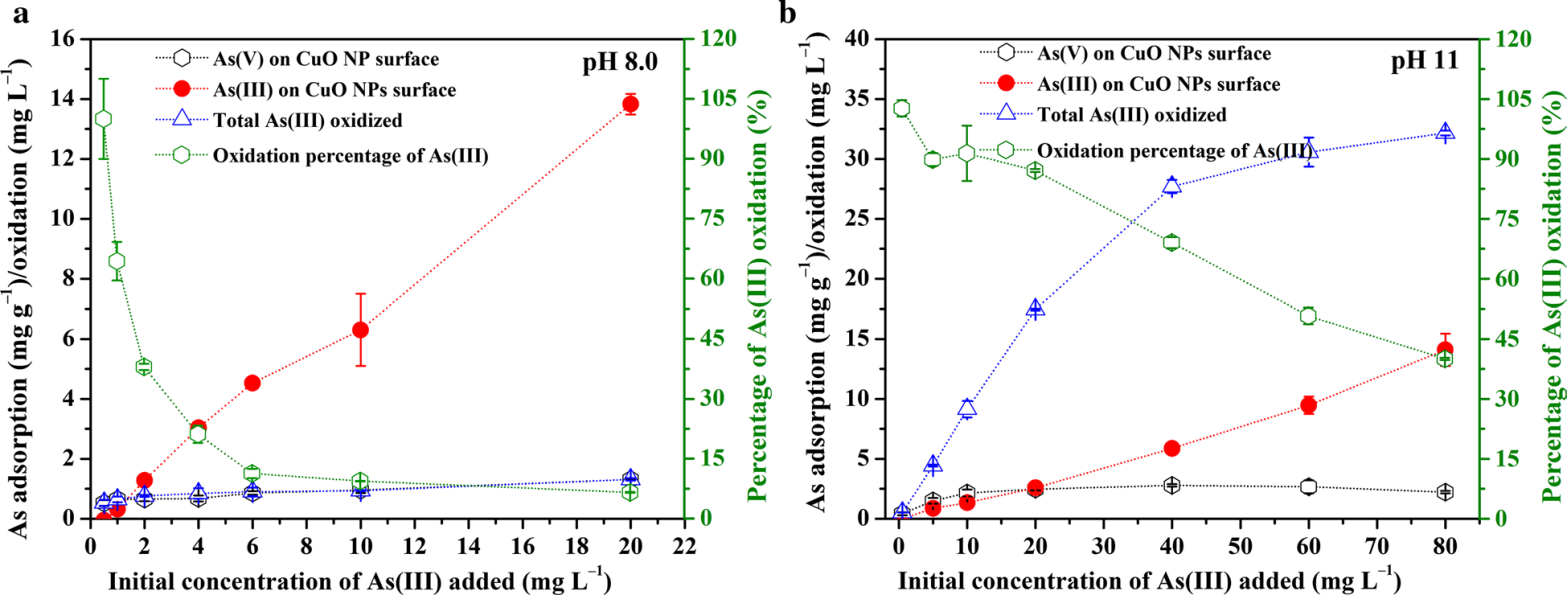
Effect of initial As(III) concentrations added on adsorption and oxidation of As(III) onto CuO NPs surfaces at pH 8 (**a**) and 11 (**b**) with 1 g L^−1^ CuO NPs

The effects of initial As(III) concentration on As(III) adsorption and oxidation were investigated at pH 8 and 11 over 24 h, and the results were shown in Fig. 3. Dramatic differences were observed between the two tested pHs: (1) at pH 8, although adsorption of As(III) increased almost linearly with initial As(III) (to 20 mg L^−1^), total oxidized As(III) plateaued rapidly (1.31 mg L^−1^), resulting in a rapid decrease in the percentage of oxidized As(III); and most of the formed As(V) remained on NPs surface; (2) at pH 11, much more As(III) was oxidized and plateaued only when the initial As(III) concentration was above 40 mg L^−1^, and the percentage of oxidized As(III) just decreased gradually; (3) although the oxidation amount increased with initial As(III) concentration, the total amount of adsorbed As(III) and As(V) was much smaller at pH 11 than at pH 8 under the same initial As(III) concentration. These results again suggest the remarkable oxidation capacity of CuO NPs at high pH.

The pH-dependent distribution of As species is most likely related to the species transformation of aqueous As(III) and As(V) and the surface charge of CuO NPs. At pH below 9, the main species of aqueous As(III) is H_3_AsO_3_, but As(V) mainly exists in the forms of H_2_AsO_4_^−^and HAsO_4_^2−^. At pH above 9, As(III) exists in the forms of H_2_AsO_3_^−^ and HAsO_3_^2−^, and aqueous As(V) as HAsO_4_^2−^ and AsO_4_^3−^ [18]. As(III) (in form of H_3_AsO_3_) is adsorbed on CuO NPs via Van der Waals force at pH 6–9 [32]. The adsorption percentage of As(V) was observed to be obviously higher than that of As(III) under the same initial As concentration at pHs ranging from 6 to 11 [25]. The aqueous As(V) in forms of H_2_AsO_3_^−^ and HAsO_3_^2−^ may have a higher affinity than As(III) to the positively charged surface of CuO NPs and tend to form inner-sphere complexes. In this study, a large number of surface reactive sites were occupied by the formed As(V), resulting in the low oxidation rate of As(III) by CuO NPs at low pHs. Due to the strongly negative surface charge of CuO NPs at high pHs, the formed As(V) (mainly in the forms of HAsO_4_^2−^ and AsO_4_^3−^) is electrostatically repulsed from CuO NPs surface. Furthermore, redox potential of As(V)/As(III) would increase with decreasing pH, indicating that As(III) is more readily oxidized at higher pH. For instance, in acid medium, the standard potential for the half reaction (H_3_AsO_4_+H^+^)/H_3_AsO_3_ is 0.56 V; but in alkaline medium, that of AsO_4_^3−^/(AsO_2_^−^+OH^−^) is − 0.71 V [33]. Therefore, CuO NPs surface remains highly reactive and can continuously oxidize As(III) at pH above the PZC.

### Q-XAS test results

**Fig. 4:**
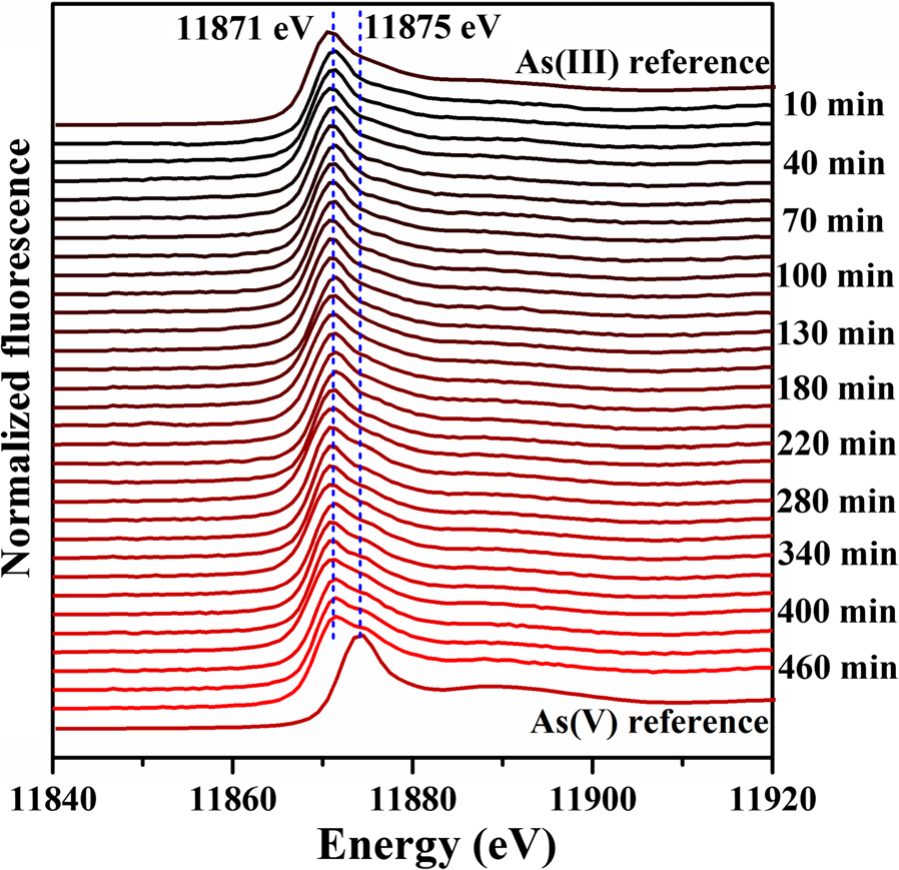
Data of individual As K-edge XANES spectra in the range from 11840 eV to 11920 eV as a function of time. Solution As(III) and As(V) was used to LCF of XANES spectra that was shown in Additional file 1: Fig. S7

In situ Q-XAS was conducted to directly monitor the oxidation of As(III) [34]. To obtain an optimal signal-to-noise ratio, we employed higher concentrations of A_S_(III) and CuO NPs for the in situ Q-XAS measurement than for batch experiments. The in situ As K-edge XANES spectra as a function of time and their LCF results are shown in Fig. 4 and Additional file 1: Fig. S7, respectively. Some data point truncations in Additional file 1: Fig. S7 were caused by the pause of synchrotron radiation facility for photon injection. In situ Q-XAS spectra over 8 h revealed that As(III) was oxidized to As(V) in CuO NPs suspension at pH 11, which is consistent with the results of batch experiments (Fig. 1c). With increasing reaction time, more As(III) was oxidized to As(V) and about 36% As(III) was oxidized after 470 min. The results of in situ Q-XAS measurement suggested that more than 10 mg As(III) could be oxidized by 1 g CuO NPs at pH 11. However, the rate constant (0.14 h^−1^) determined in batch experiments at this pH was much higher than that determined in Q-XAS experiment (0.03 h^−1^), which is probably due to the difference in the initial As(III) concentrations used in the two experiments. It is also possible that aggregation of CuO NPs at higher solute concentration may inhibit the oxidation of As(III) [35].

### XPS analyses

**Fig. 5:**
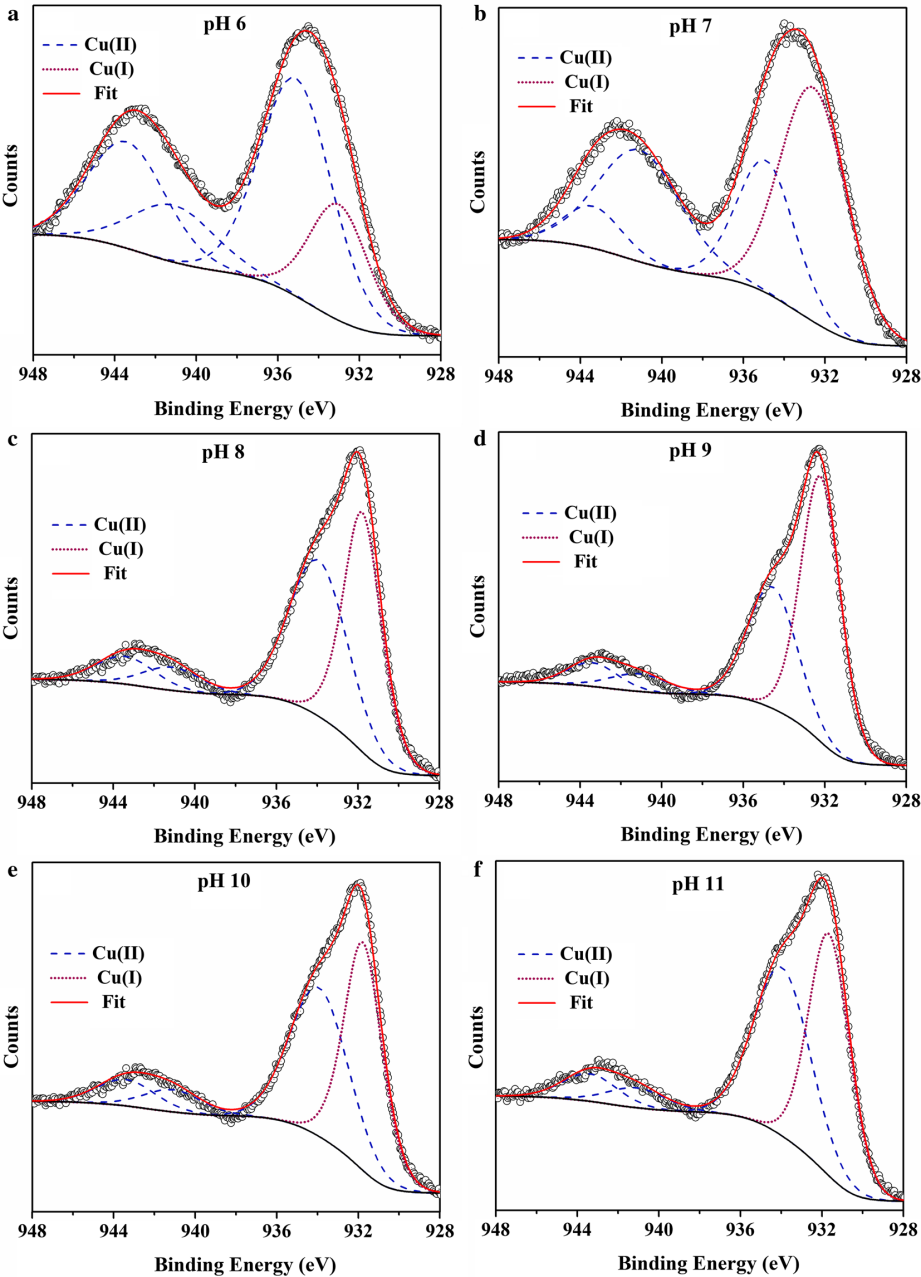
High-resolution Cu2p spectra and their fits of CuO NPs samples after 48 h reaction with As(III) under the same As(III) concentration (10 mg L^−1^) at pH 6 (**a**), 7 (**b**), 8 (**c**), 9 (**d**), 10 (**e**), 11 (**f**) in the open system

High-resolution Cu2p and As3d spectra of pure and reacted CuO NPs are shown in Fig. 5, Additional file 1: Figures S5, S6. The peak at 933.0 eV could be assigned to the binding energy of Cu(I) species, and the peaks at 934.8, 941.1, and 943.4 eV might belong to the binding energy of Cu(II) species [17, 36]. The surface Cu compositions of these selected samples determined by XPS fitting are presented in Additional file 1: Table S1. At higher pH, the proportion of Cu(I) tended to increase, implying that some Cu(II) was transformed to Cu(I) at the near-surface of CuO NPs. Given that CuO is likely reduced during XPS measurement [17, 36], the measured content of Cu(I) can only reflect its relative composition on the surface. Moreover, another reason for the presence of Cu(I) species might be the reduction of a small amount of Cu(II) by glacial acetic acid added during the synthesis of CuO NPs [37]. A large amount of Cu(II) was present on the surface, indicating that Cu(I) might not be the final stable form during As(III) oxidation, but an intermediate phase involved in As(III) oxidation. Due to the relatively low initial As(III) concentration, the amount of As on CuO NPs surface was not sufficient for the collection of As3d spectra with a good noise-signal ratio Additional file 1: Fig. S6. However, the results showed that the intensity of As3d spectra at pH below PZC was higher than that at pH above PZC, which further confirms the negative effect of pH on As adsorption. The binding energy of As3d for As(V) was about 1 eV greater than that for As(III) [38, 39], which can help to distinguish As(III) and As(V). As3d spectra in the tested samples of pH 6, 7 and 8 showed no clear energy shift, and exhibited a slight energy shift at pH 9 (Additional file 1: Fig. S6). The spectral intensity was too weak for the observation of any change in energy shift in pH 10 and 11 samples, probably due to the small adsorption amounts of As at these pHs.

### EPR analysis for ROS

**Fig. 6:**
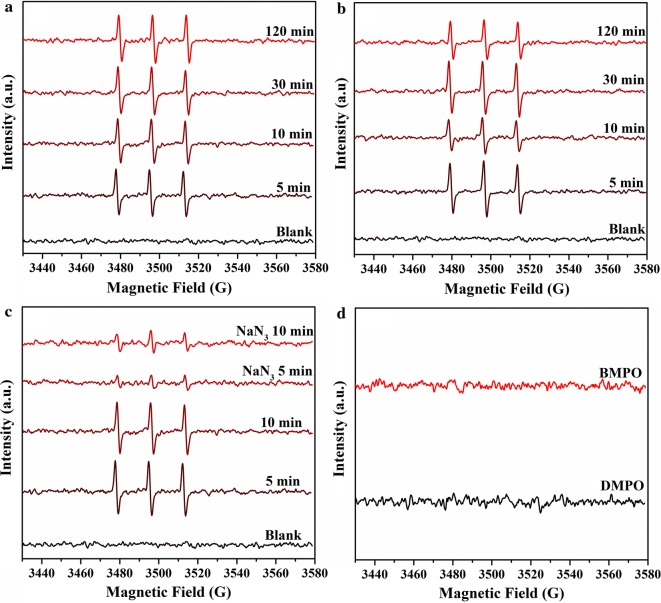
EPR spectra of CuO NPs suspension in the presence of TEMP without As(III) (**a**) and with the addition of As (III) at pH 11.0 (**b**); EPR spectra of CuO NPs suspension in the presence of TEMP after the reaction with and without NaN_3_ (**c**) and in the presence of DMPO and BMPO (**d**) without As(III) at pH 11

In systems containing CuO NPs, ROS is commonly generated by Fenton-like heterogeneous reactions through the Cu(II)/Cu(I) redox couple at the interfaces [40]. EPR experiments were conducted to reveal the production and speciation of ROS in both pure and reaction systems at the selected reaction intervals. As a spin trap, TEMP can specifically capture ^1^O_2_ to form TEMPONE, a nitroxide radical with a stable EPR signal [41]. Figure 6a, b show the EPR spectra of TEMPONE in the absence and presence of As(III) for different reaction time periods. The EPR spectra clearly reveal that ^1^O_2_ was consistently produced in all pure CuO NPs suspensions (Fig. 6a). The EPR signal intensity of TEMPONE did not vary temporally (Fig. 6a), suggesting a stable generation and accumulation of ^1^O_2_ in the suspension during the whole process. The addition of 10 mM NaN_3_, a ^1^O_2_ quencher, substantially decreased the ^1^O_2_ EPR signal (Fig. 6c), further verifying the production of ^1^O_2_ species in CuO NPs suspensions at pH 11. After the addition of As(III) into CuO NPs suspension, the EPR signal intensity of TEMPONE was decreased at 10 min, and then restored to the normal level at 30 min. At 120 min, the EPR signal intensity dropped again (Fig. 6b). The instability of ^1^O_2_ EPR signal was possibly caused by the reaction between As(III) and ^1^O_2_ species, and continuous production of ^1^O_2_ in the reaction (which will be discussed below). No DMPO-OOH, DMPO-OH, BMPO-OOH and BMPO-OH adducts were detected in EPR measurements of the CuO NPs suspension (Fig. 6d). In combination with macroscopic analyses, ^1^O_2_ might play a crucial role in the oxidation of As(III) by CuO NPs. Additionally, the presence and impacts of other ROS species, such as ·OH and O_2_·^−^, could not be determined, though they may also be involved in the reaction.

### As(III) oxidation mechanisms

Based on the first sharp increase and the subsequent slow decrease of As(III) on CuO NPs surface (Fig. 1), we propose that As(III) might be firstly adsorbed on the surface of CuO NPs and then slowly oxidized to As(V). Therefore, at pH below PZC, As(III) and the produced As(V) are mainly adsorbed on the surface of CuO NPs; at pH above PZC, the adsorbed As(III) can be rapidly oxidized to As(V) on the surface and then the produced As(V) is abruptly desorbed into the solution from CuO NPs surface, which ensures that an active surface is available for further oxidation of newly adsorbed As(III).

**Fig. 7:**
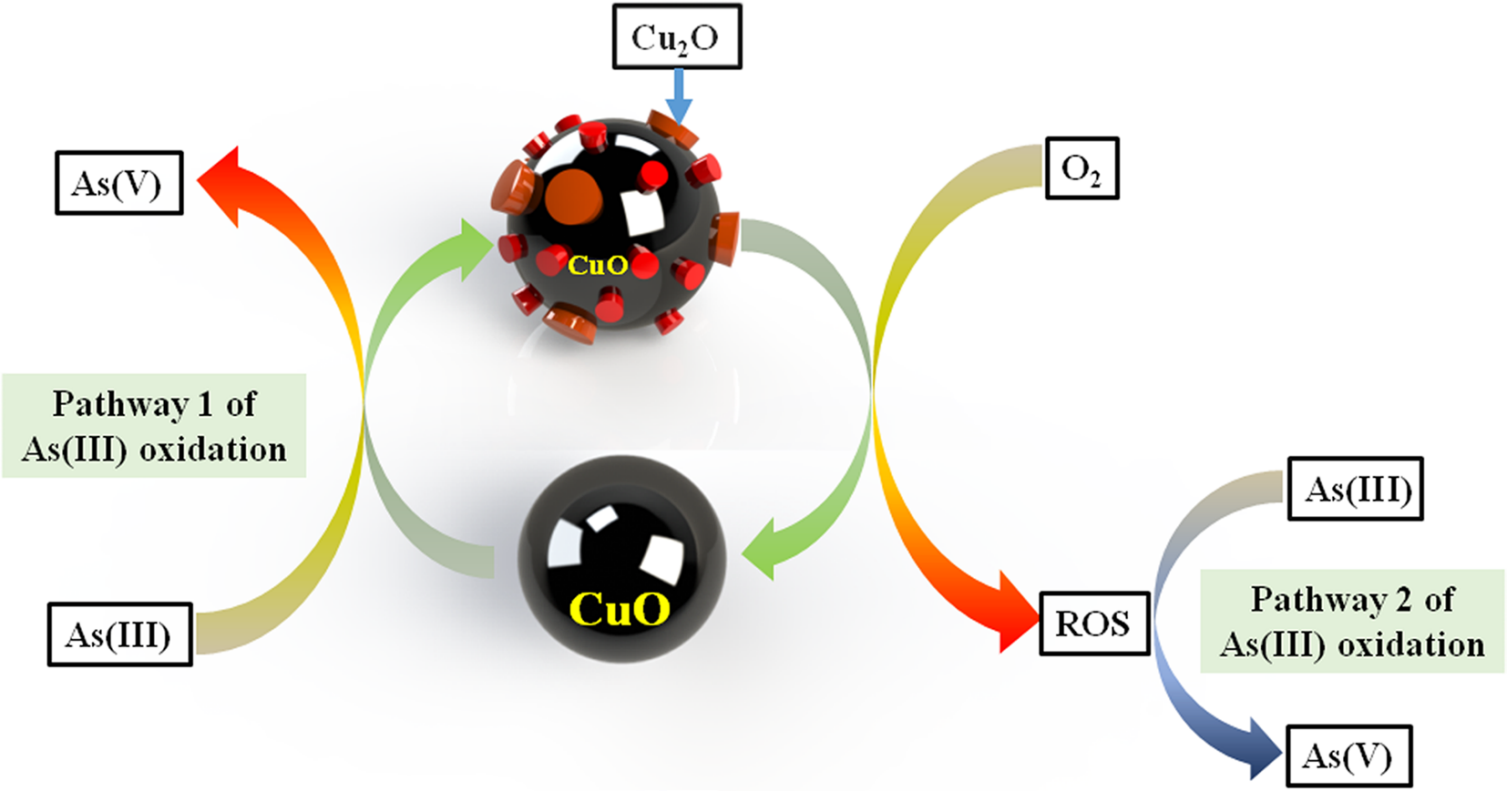
The proposed pathways of arsenite catalytic oxidation on CuO NPs surface

Our XPS data indicated that part of Cu(II) was converted to Cu(I) at the near-surface (Fig. 5). It could be inferred that the oxidation of some As(III) was triggered by the conversion of CuO to Cu_2_O (Eq. 1) at the electron donor active sites on CuO NPs [10]. It has been suggested that the number of these active sites sharply rises when the particle size decreases down to nano-scale [42]. The particle size of CuO NPs used in this study was around 15 nm in width and 60 nm in length, which could contribute to a high surface activity. Generally, Cu_2_O is not stable in open systems and is readily re-oxidized to CuO by dissolved O_2_ [43], resulting in the continuous catalytic oxidation of As(III) on the surface of CuO NPs (Eqs. 2 and 3), which can be regarded as the first pathway of As(III) oxidation (Fig. 7).

In addition, the Cu(I) in CuO NPs could be responsible for the production of ROS [28]. O_2_·^−^ could be produced on the reactive site with electron transfer from Cu(I) to O_2_. However, O_2_·^−^ was not detected using EPR in our study. It is possible that O_2_·^−^ was not a stable species under our experimental conditions, and its concentration was too low to be detected. In fact, O_2_·^−^ is readily transformed to ^1^O_2_ via disproportionation reaction [44], which was detected in CuO NPs suspension at the absence or presence of As(III) (Fig. 6). The standard redox potential of ^1^O_2_/H_2_O is 2.204 eV [45], indicating that ^1^O_2_ is a strong oxidant similar to ·OH, which has a standard redox potential of 2.538 eV (·OH/H_2_O). Therefore, we suggest the second reaction pathway: As(III) is oxidized by ROS produced via the activation of O_2_ on Cu(I) sites of the CuO NPs surface (Fig. 7). CuO will be re-generated from Cu(I) after reaction with O_2_, and participates in the further oxidation of As(III) via the above-mentioned first reaction pathway. Figure 1d shows that dissolved O_2_ is an essential factor for the high rate of As(III) oxidation by CuO NPs. Formation of ^1^O_2_ from O_2_ in the presence of CuO NPs might also contribute to As(III) oxidation by CuO NPs (Eqs. 3 and 4). The observation of continuous ^1^O_2_ production in the experiments (Fig. 6) indicates a close coupling of these two pathways, which can explain the cycling of CuO NPs catalyst and efficient As(III) oxidation. That is to say, the oxidation of As(III) by the re-generated Cu(II) active sites via the first pathway yields Cu(I) sites again, which could trigger the continuous production of ^1^O_2_.

In addition, titration experiment was performed to compare the differences in the amount of consumed OH^−^ between the N_2_ and open systems at pH 11. In the N_2_ system, only 0.022 mmol OH^−^ was consumed at the very beginning, which might result from the surface hydrolysis of CuO NPs. However, in the open system, OH^−^ consumption gradually increased from 0.022 mmol at 2 h to 0.080 mmol at 12 h as the reaction proceeded (Additional file 1: Fig. S8). These results suggest that H^+^ production or OH^−^ consumption is associated with As(III) oxidation.

Taking the above results together, we propose the reaction pathways of As(III) oxidation at high pH as the following equations.

1 $${\text{As}}\left( {\text{III}} \right) + 2CuO \to As\left( V \right) + Cu_{2} O$$

2 $$2Cu_{2} O + O_{2} \to 4CuO$$

3 $$O_{2} \mathop {\longrightarrow} \limits^{{CuO/Cu_{2} O }} {}_{{}}^{1} O_{2}$$

4 $$2{\text{CuO}} \equiv H_{2} AsO_{3} + {}_{{}}^{1} O_{2} + 2OH^{ - } \to 2CuO \equiv HAsO_{4}^{ - } + 2H_{2} O$$

In general, ROS is mainly formed in acidic solution, and Cu^2+^ cations in solution play an important role in ROS production [26–28]. But the above XPS results indicate that the changes of Cu valence mainly occur at pH > 9, when Cu^2+^ could not be present in solution (Additional file 1: S4). Therefore, ROS is probably formed via electron transfer from Cu(I) to O_2_ on the surface of CuO NPs. Even when O_2_ is absent in the system, direct electron transfer from As(III) to Cu(II) can occur on the surface of CuO NPs. These findings can also facilitate a better understanding about the impact of CuO NPs on the mobility and transformation of some redox-sensitive substances in various geochemical settings.

## Conclusions

Our results verify that CuO NPs is capable to catalytically oxidize As(III) efficiently with dissolved O_2_ as the terminal electron acceptor using in situ spectroscopic techniques (Fig. 4). Therefore, CuO NPs can be a potential catalyst and adsorbent to affect the geochemical behaviors of As, which can help researchers to predict the risk of CuO NPs before the application of it in some industrial and environmental fields. Also, this study provides a new perspective for investigation of As(III) oxidation process related to Cu-based NPs.

It can be indicated that the amount and rate of As(III) oxidation by CuO NPs are greatly enhanced by increasing pH to a high alkaline range (Fig. 3b). It should be noted that the adsorption of produced As(V) decreases to a certain degree in alkaline solution. Thus, other adsorbents with high As(V) retention ability at alkaline pHs could be applied simultaneously to enhance the removal of aqueous As to meet the environmental standard in As contaminated areas. Besides, it will be of great environmental significance to further improve the catalytic oxidation capacity of CuO NPs at around neutral pHs. Actually, our undergoing study has shown that the addition of aqueous Mn(II) could remarkably enhance the oxidation of As(III) and the adsorption of As(V) at near neutral pH (Additional file 1: Fig. S9).

Furthermore, two coupled reaction pathways, i.e. direction oxidation by CuO and oxidation by ROS produced via O_2_ activation on Cu(I) surface sites, are proposed for As(III) oxidation (Fig. 7). These findings further demonstrate the high catalytic activity of CuO NPs towards the oxidation reactions in water, implying the important role of CuO NPs to affect the fate and geochemical behaviors of some reducing pollutants and redox-sensitive organic substances in the environment. The stability and potential reusability of CuO NPs also make it an ideal candidate to be applied in permeable reactive barrier (PRB) in ground water purification. Actually, some developing countries (e.g., Bangladesh and West Bengal of India [46]) suffer from heavy As contamination in the groundwater, and usually there is a serious lack of water treatment facilities to purify the As-contaminated groundwater [47, 48]. Systematic studies of the adsorption–oxidation mechanisms of As(III) on CuO NPs surfaces are significant for a full understanding of the potential influence of CuO NPs on the reductive pollutants, and for the further development of reliable techniques to deal with As contamination efficiently.

## Additional file

**Additional file 1: S1.** Reagents. **S2**. Preparation and characterizations of CuO NPs. **S3**. Q-XAS experimental details. **S4**. EPR spectroscopy detection procedures. **S5**. CuO NPs characterizations. **Fig. S1**. The XRD pattern (**a**), TEM image (**b**), FTIR spectra (**c**), and Zeta potential data (**d**) of synthesized CuO NPs. **Fig. S2**. Fits of pseudo first-order model of As(III) oxidation kinetic at pH 6 (**a**), pH 9 (**b**), and pH 11 (**c**) in the open system, and at pH 11 in the N_2_ atmosphere (**d**). The fitting parameters were showed in Table 1. **Fig. S3**. Kinetics of As(III) oxidation in deionized water, in presence of Cu(OH)_2_ and in presence of CuO at pH 11, respectively. This experiment was set to verify that As(III) oxidation can not occur without CuO addition at pH 11. **Fig. S4**. Kinetics of Cu^2+^ released at pH 7, pH 8 and pH 11 in the open system during the As(III) adsorption and oxidation reaction, with initial As(III) of 10 mg L^−1^ and CuO NPs of 1 g L^−1^. **Fig. S5.** High-resolution Cu2p spectra and their fits of raw CuO NPs. **Fig. S6.** High-resolution As3d spectra CuO NPs after reaction with As(III) under same As(III) concentration (10 mg L^−1^) at pH 6, 7, 8, 9, 10 and 11 in the open system. **Fig. S7.** The concentration and percentage of As(V) and As(III) determined from the LCF of quick As XANES, the initial As(III) concentration is 150 mg L^−1^. **Fig. S8.** The volume of NaOH (0.1 M) consumed and Eh variation during As(III) (10 mg L^−1^) with CuO NPs at pH 11 in the open system or N_2_ system. **Fig. S9.** Kinetics of As(III) oxidation on CuO NPs surface with adding of Mn(II) at pH 8 in the open system. The initial mol Mn(II)/As(III) = 6 is designed (10 mg L^−1^ initial As(III)). **Scheme S1.** Experimental setup used to collect Q-XAS data. **Table S1.** Fitting parameters used for Cu (3d) spectra of samples at different pHs.
