# Supplementary material for: Catalytic oxidation of arsenite and reaction pathways on the surface of CuO nanoparticles at a wide range of pHs
Source: Geochem Trans. 2018 Jun 22;19:12. doi: 10.1186/s12932-018-0058-3 (PMC6014938; doi:10.1186/s12932-018-0058-3)
Supplement: Supplementary file 1 — Additional file 1: S1. Reagents. S2. Preparation and characterizations of CuO NPs. S3. Q-XAS experimental details. S4. EPR spectroscopy detection procedures. S5. CuO NPs characterizations. Fig. S1. The XRD pattern (a), TEM image (b), FTIR spectra (c), and Zeta potential data (d) of synthesized CuO NPs. Fig. S2. Fits of pseudo first-order model of As(III) oxidation kinetic at pH 6 (a), pH 9 (b), and pH 11 (c) in the open system, and at pH 11 in the N2 atmosphere (d). The fitting parameters were showed in Table 1. Fig. S3. Kinetics of As(III) oxidation in deionized water, in presence of Cu(OH)2 and in presence of CuO at pH 11, respectively. This experiment was set to verify that As(III) oxidation can not occur without CuO addition at pH 11. Fig. S4. Kinetics of Cu2+ released at pH 7, pH 8 and pH 11 in the open system during the As(III) adsorption and oxidation reaction, with initial As(III) of 10 mg L−1 and CuO NPs of 1 g L−1. Fig. S5. High-resolution Cu2p spectra and their fits of raw CuO NPs. Fig. S6. High-resolution As3d spectra CuO NPs after reaction with As(III) under same As(III) concentration (10 mg L−1) at pH 6, 7, 8, 9, 10 and 11 in the open system. Fig. S7. The concentration and percentage of As(V) and As(III) determined from the LCF of quick As XANES, the initial As(III) concentration is 150 mg L−1. Fig. S8. The volume of NaOH (0.1 M) consumed and Eh variation during As(III) (10 mg L−1) with CuO NPs at pH 11 in the open system or N2 system. Fig. S9. Kinetics of As(III) oxidation on CuO NPs surface with adding of Mn(II) at pH 8 in the open system. The initial mol Mn(II)/As(III) = 6 is designed (10 mg L−1 initial As(III)). Scheme S1. Experimental setup used to collect Q-XAS data. Table S1. Fitting parameters used for Cu (3d) spectra of samples at different pHs. [file 12932_2018_58_MOESM1_ESM.doc]

***Supplementary Material***

**Catalytic Oxidation of Arsenite and reaction pathways at the Surfaces of CuO Nanoparticles at a wide range of pHs**

Lingqun Zeng^a#^, Biao Wan^a#^, Rixiang Huang^b^, Yupeng Yan^a^, Xiaoming Wang^a^, Wenfeng Tan^a^, Fan Liu^a^, Xionghan Feng^a^*

^a^ Key Laboratory of Arable Land Conservation (Middle and Lower Reaches of Yangtze River), Ministry of Agriculture, College of Resources and Environment, Huazhong Agricultural University, Wuhan 430070, China

^b^ School of Earth and Atmospheric Sciences, Georgia Institute of Technology, 311 Ferst Dr, Atlanta, GA 30324-0340, USA

^#^ The authors contribute equally to this work

Lingqun Zeng: zlq91@webmail.hzau.edu.cn

Biao Wan: wanbiao@gatech.edu

Rixiang Huang: rixiang.huang@eas.gatech.edu

Yupeng Yan: ypyan@mail.hzau.edu.cn

Xiaoming Wang: [wangxm338@mail.hzau.edu.cn](mailto:wangxm338@mail.hzau.edu.cn)

Wenfeng Tan: tanwf@mail.hzau.edu.cn

Fan Liu: liufan@mail.hzau.edu.cn

Xionghan Feng: [fxh73@mail.hzau.edu.cn](mailto:fxh73@mail.hzau.edu.cn)

*Corresponding author: Xionghan Feng; Tel: +86 27 87280271; Fax: +86 27 87288618. E-mail address: [fxh73@mail.hzau.edu.cn](mailto:fxh73@mail.hzau.edu.cn) (X. Feng).

**S1. Reagents.**

Cu(NO_3_)_2_·3H_2_O, Arsenic trioxide (As_2_O_3_), NaOH, and HCl were purchased from Sinopharm Chemical Reagent Co., Ltd, China; 2, 2, 6, 6-Tetramethylpiperidine (TEMP) were purchased from TCI Shanghai Chemical Industry Co., Ltd.; 5, 5-dimethyl-1-pyrroline N-oxide (DMPO, for ESR) were purchased from Sigma-Aldrich, Inc (St Louis, MO, USA); 3, 4-dihydro-2-methyl-1,1-dimethylethyl ester-2H-pyrrole-2-carboxylic acid-1-oxide (BMPO) were purchased from Cayman Chemical, Inc (Ellsworth Road Ann Arbor, MI, USA). All chemical reagents were of analytical grade. Stock solution containing 13.33 mM As(III) (1.0 g L^−1^) was prepared by dissolving 1.3186 g of As_2_O_3_ in 500 mL of DI water containing 2% (w/v) NaOH. Deionized water (Aquapro 2 s, with resistivity of 18.25 MΩ cm^−1^ at 25 ºC) was used for the preparation of solutions and rinsing processes.

**S2. Preparation and characterizations of CuO NPs**

In a typical procedure, a set of 300 mL of 0.02 M Cu(NO_3_)_2_ solution was prepared by dissolving analytically pure Cu(NO_3_)_2_·3H_2_O in deionized water. The set of solution was added into a flask, equipped with a refluxing device. The flask was kept at temperature of 100 ◦C with vigorous stirring, in which 1 mL glacial acetic acid was added into the Cu(NO_3_)_2_ solution. Then 0.50 g of solid NaOH (platelets) was rapidly added into solution, where a large amount of blue or black precipitate was simultaneously produced and maintained at the crystallization temperature for 20 min. Next, the precipitate was heated at the same temperature for another 20 min. After all reactions were completed, the resulting products were centrifuged, washed with water and ethanol for several times and dried in air at room temperature.

The synthesized product was identified by XRD using a Bruker D8 ADVANCE X-ray diffractometer (Bruker AXS Gmbh, Karlsruhe, Germany), and its morphology and particle size were determined by high-resolution transmission electron microscopy (HRTEM) (Philips, Eindhoven, the Netherlands). Its point of zero charge (PZC) was measured using a zeta potential analyzer (Malvern Zetasizer ZEN 3000, UK). The specific surface area was determined by five-point Brunauer-Emmett-Teller (BET) N_2_ adsorption method (Quantichrome Corp.). Fourier-transformed infrared spectroscopy (FT-IR) was performed on a Bruker Vertex 70 FTIR spectrometer (Bruker Equinox 55 type). CuO and KBr were mixed with gram ratio of 1:100. The 256 scans were measured in the spectral range of 3500-400 cm^−1^ with a resolution of 4 cm^−1^.

**S3. Q-XAS experimental details**

The procedures and methods of data collection can be found in Ginder-Vogel et al. (2009) . The molar ratio of As(III) and As(V) was determined via Linear Combination Fitting (LCF) of the collected XANES data using the XAS spectra of 6.67 mM As(III) and 6.67 mM As(V) solutions as standard references. The XAS data analysis was performed using the ATHENA from IFEFFIT program package (version 1.2.11).

**S4. EPR spectroscopy detection procedures**

2 mM spin trapping agent 2, 2, 6, 6-Tetramethylpiperidine (TEMP) was used for detection of singlet oxygen (^1^O_2_). 25 mM 3, 4-dihydro-2-methyl-1, 1-dimethylethylester-2H-pyrrole-2-carboxylic acid-1-oxide (BMPO) and 10 mM 5, 5-dimethyl-1-pyrroline N-oxide (DMPO) were used for detecting hydroxyl radicals (•OH) and superoxide (O_2_•^−^), respectively. To further confirm the speciation of the produced ROS, the effects of 10 mM NaN_3_ were investigated as typical scavengers for ^1^O_2_. All EPR measurements were done using a Bruker X-band A200 Electron-Spin Resonance spectrometer at ambient temperature. The EPR experimental conditions were as follows: resonance frequency, 9.81 GHz; microwave power, 22.08 mW; modulation frequency, 100 kHz; modulation amplitude, 1.0 G; sweep width, 200 G; time constant, 163.83 ms; sweep time, 40.96 s; and receiver gain, 1.0×10^4^.

**S5. CuO NPs characterizations**

XRD pattern indicates that the synthesized CuO NPs is pure and match with single phase monoclinic CuO standard pattern (JCPDS No. 00-001-1142) (Figure S1a). The synthesized CuO NPs is shuttle-like shape with about 15 nm in width and 60 nm in length (Figure S1b) and its specific surface area is 79 m^2^ g^-1^. The IR bands at 526, 1016, 1525, 3419 cm^-1^ in Figure S1c can be attributed to the stretching vibrations of Cu−O, C−O, C−O and O−H groups , respectively. The zeta potential of CuO NPs decreases with the increase of solution pHs (Figure S1d) with PZC at ~9.2, similar to reported results .


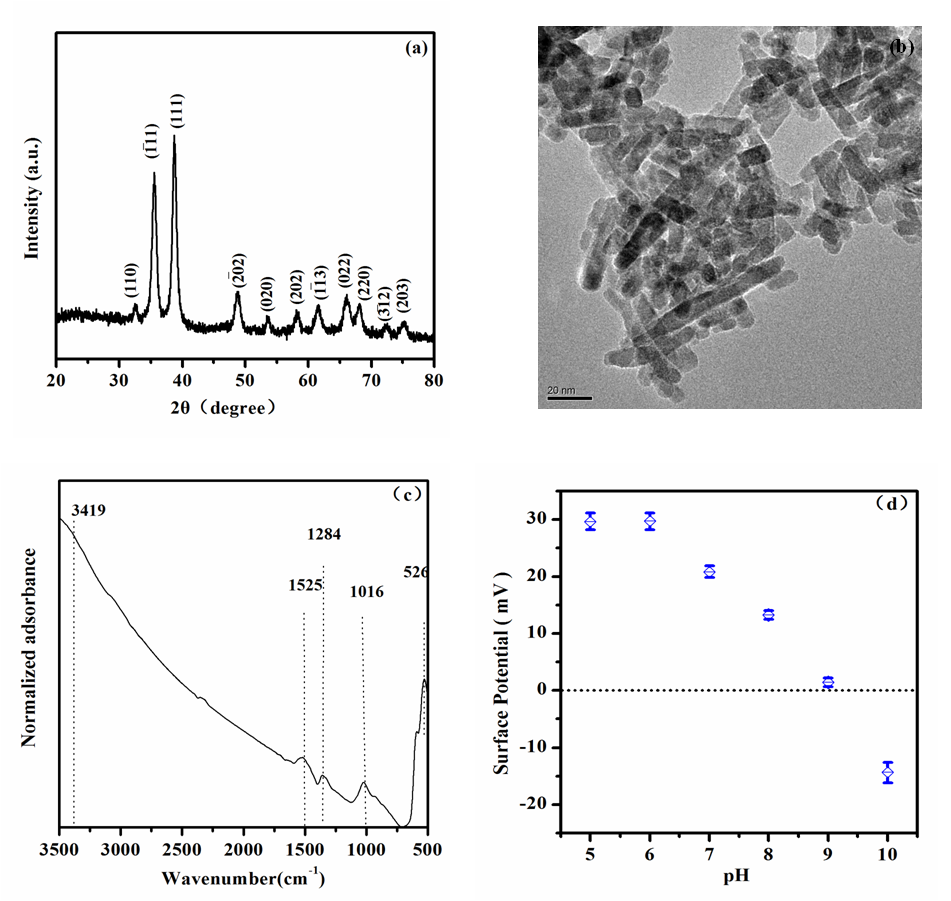


**Fig. S1**. The XRD pattern (a), TEM image (b), FTIR spectra (c), and Zeta potential data (d) of synthesized CuO NPs.

A





**Fig. S2.** Fits of pseudo first-order model of As(III) oxidation kinetic at pH 6 (a), pH 9 (b), and pH 11 (c) in the open system, and at pH 11 in the N_2_ atmosphere (d). The fitting parameters were showed in Table 1.





**Fig. S3.** Kinetics of As(III) oxidation in deionized water, in presence of Cu(OH)_2_ and in presence of CuO at pH 11, respectively. This experiment was set to verify that As(III) oxidation can not occur without CuO addition at pH 11.

**

**

**Fig. S4.** Kinetics of Cu^2+^ released at pH 7, pH 8 and pH 11 in the open system during the As(III) adsorption and oxidation reaction, with initial As(III) of 10 mg L^−1^ and CuO NPs of 1 g L^−1^.

**
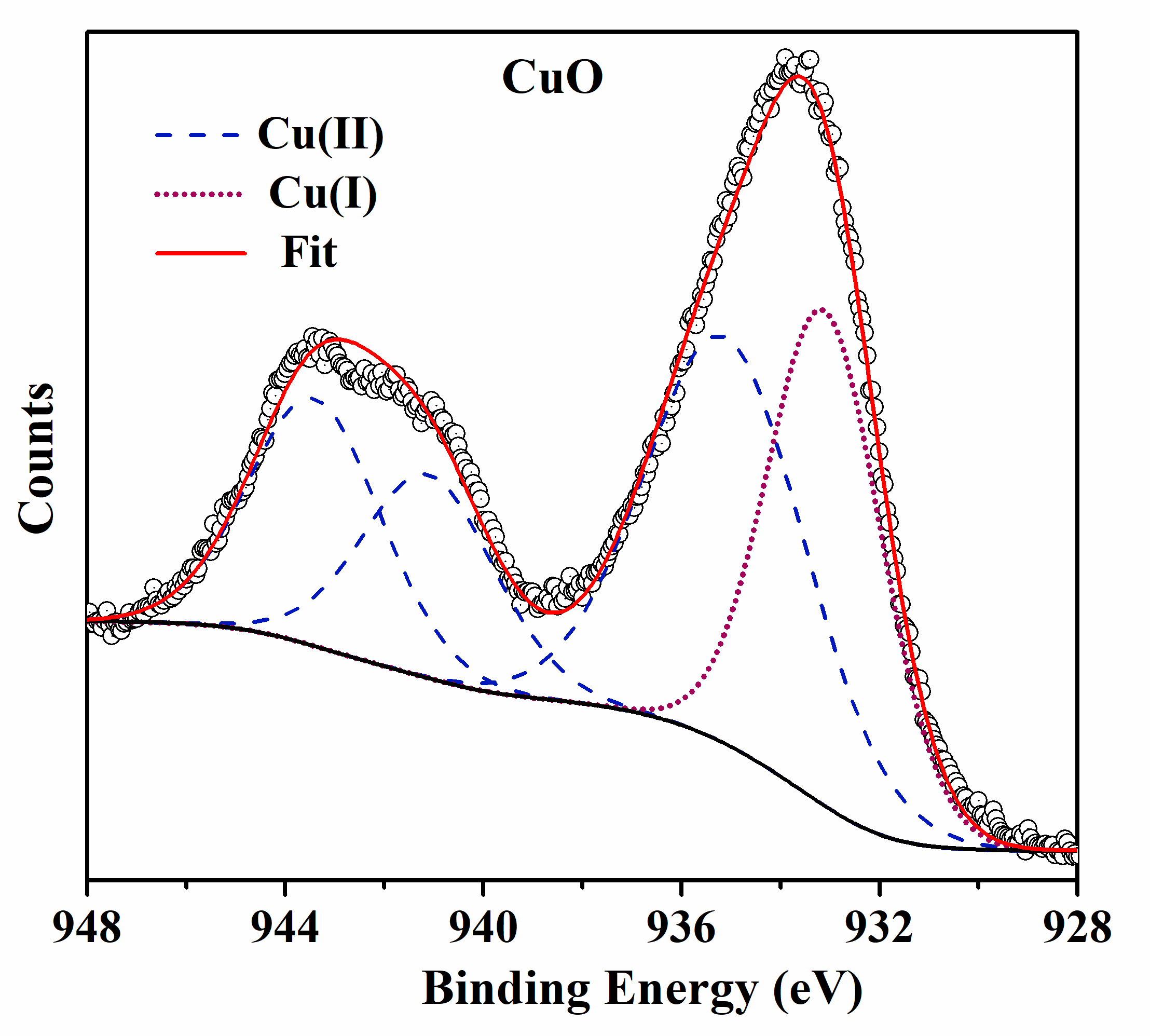
**

**Fig. S5.** High-resolution Cu2p spectra and their fits of raw CuO NPs.

**

**

**Fig. S6.** High-resolution As3d spectra CuO NPs after reaction with As(III) under same As(III) concentration (10 mg L^-1^) at pH 6, 7, 8, 9, 10 and 11 in the open system.

**

**

**Fig. S7.** The concentration and percentage of As(V) and As(III) determined from the LCF of quick As XANES, the initial As(III) concentration is 150 mg L^−1^.


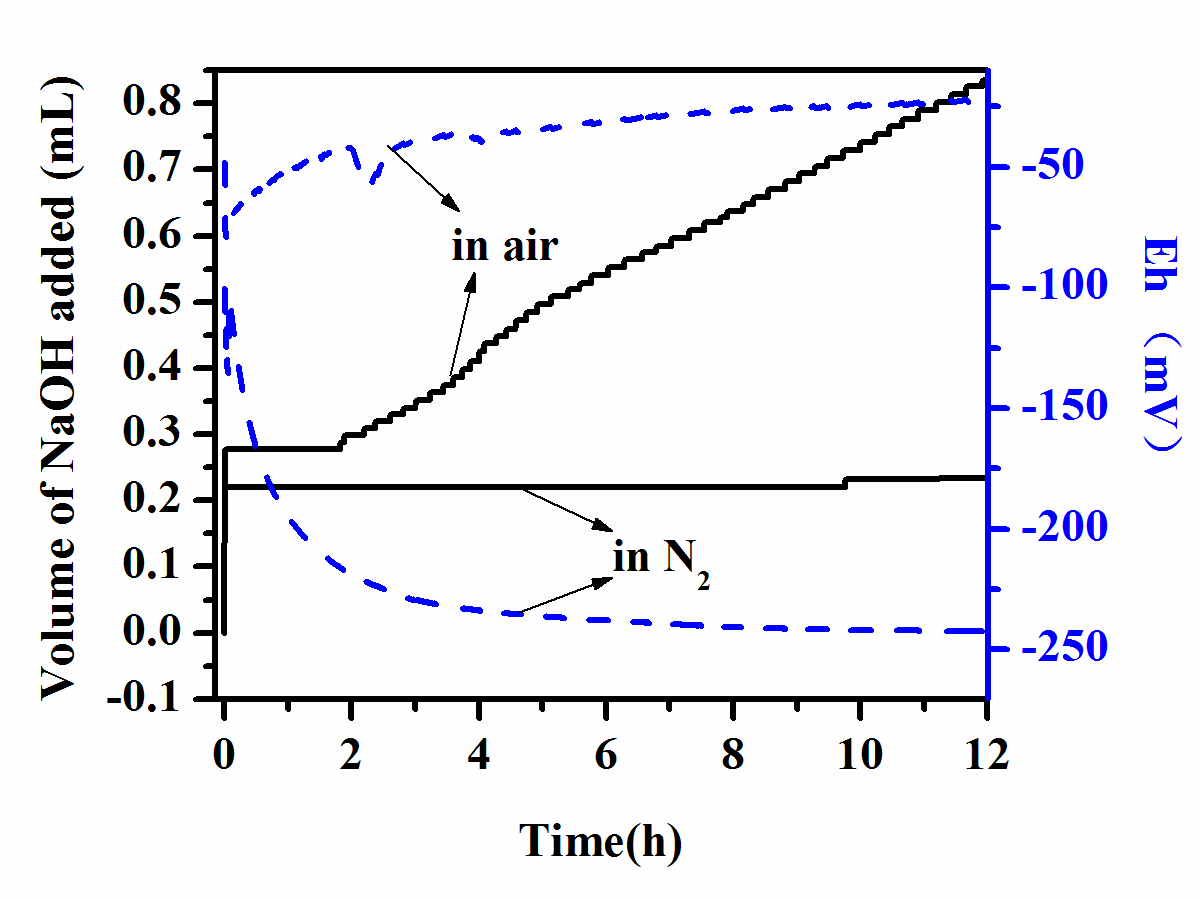


**Fig. S8.** The volume of NaOH (0.1 M) consumed and Eh variation during As(III) (10 mg L^−1^) with CuO NPs at pH 11 in the open system or N_2_ system.





**Fig. S9.** Kinetics of As(III) oxidation on CuO NPs surface with adding of Mn(II) at pH 8 in the open system. The initial mol Mn(II)/As(III)=6 is designed (10 mg L^−1^ initial As(III)).

Lytle Fluorescence

Detector (I_f_)

X-rays

X-rays

Gas Ionization Chamber (I_0_)

Magnetic Stir Plate linking to automatic titrator

**Scheme S1**. Experimental setup used to collect Q-XAS data.

**Table S1**. Fitting parameters used for Cu (3d) spectra of samples at different pHs.

| **Samples of CuO** | **Species** | **BE(eV)** | **FWHM(eV)** | **At.%** |
| --- | --- | --- | --- | --- |
| Intact | Cu(II) | 943.4 | 3.5 | 69.49 |
|  |  | 941.1 | 3.5 |  |
|  |  | 935.1 | 3.96 |  |
|  | Cu(I) | 933.0 | 2.73 | 30.51 |
| React at pH 6 | Cu(II) | 943.4 | 4.75 | 82.14 |
|  |  | 941.1 | 5 |  |
|  |  | 935.1 | 4.3 |  |
|  | Cu(I) | 933.0 | 3.4 | 17.86 |
| React at pH 7 | Cu(II) | 943.4 | 3.5 | 67.01 |
|  |  | 941.1 | 3.25 |  |
|  |  | 934.8 | 3.48 |  |
|  | Cu(I) | 933.25 | 3.07 | 32.99 |
| React at pH 8 | Cu(II) | 943.4 | 3.5 | 57.63 |
|  |  | 941.1 | 4.94 |  |
|  |  | 934.9 | 3.5 |  |
|  | Cu(I) | 932.56 | 3.88 | 42.37 |
| React at pH 9 | Cu(II) | 943.4 | 3.5 | 55.16 |
|  |  | 941.1 | 5 |  |
|  |  | 934.9 | 3.5 |  |
|  | Cu(I) | 932.56 | 4.08 | 44.84 |
| React at pH 10 | Cu(II) | 943.4 | 3.5 | 57.08 |
|  |  | 941.1 | 3.5 |  |
|  |  | 933.91 | 3.5 |  |
|  | Cu(I) | 931.77 | 2.34 | 42.92 |
| React at pH 11 | Cu(II) | 943.4 | 3.28 | 54.75 |
|  |  | 941.1 | 3.5 |  |
|  |  | 933.96 | 3.5 |  |
|  | Cu(I) | 931.67 | 2.49 | 45.25 |

BE: binding energy FWHM: full width at half maximum

**References**

1. Ginder-Vogel M, Landrot G, Fischel JS, Sparks DL (2009) Quantification of rapid environmental redox processes with quick-scanning x-ray absorption spectroscopy (Q-XAS). *Proc* *Natl Acad Sci USA* 106:16124-16128.

1. Goswami A, Raul PK, Purkait MK, Arsenic adsorption using copper (II) oxide nanoparticles (2012) *Chem Eng Res Desi* 90:1387-1396.

3. Yoon RH, Salman T, Donnay G (1979) Predicting points of zero charge of oxides and hydroxides. *J Colloid Interf Sci* 70:483-493.
